# Empathy of Marine Aquaculture on Aquatic Products Consumption

**DOI:** 10.3389/fpsyg.2022.902889

**Published:** 2022-07-12

**Authors:** Le Xilin, Yingqi Wu, Yan Zeng, Ting Ma, Yating Wang, Qingyu Zhang

**Affiliations:** ^1^School of Economics and Management, Nanchang Institute of Science and Technology, Nanchang, China; ^2^College of International Economics and Trade, Jiangxi University of Finance and Economics, Nanchang, China; ^3^School of Government, Shenzhen University, Shenzhen, China; ^4^Key Laboratory of Regional Sustainable Development and Modelling, Institute of Geographic Sciences and Natural Resources Research, Chinese Academy of Sciences, Beijing, China; ^5^Research Institute of Business Analytics and Supply Chain Management, College of Management, Shenzhen University, Shenzhen, China

**Keywords:** empathy marketing, green marketing, perceived behavior control, sustainable consumption, post COVID-19 era

## Abstract

The prolonged COVID-19 has caused a global lockdown and greatly impacts the supply chain and consumers’ consumption behavior of aquatic products. Specifically, consumers’ increasing awareness of environmental protection drives the marine aquaculture enterprises to supply green products and establish empathy with consumers in a bid to achieve sustainable consumption. This paper conducts a study on the international green marketing paths of marine aquatic products through PLS-SEM analysis of the 407 valid samples collected from the questionnaire survey. The findings indicate that empathy marketing has a significant positive effect on consumers’ purchase intention; subjective norm has a significant positive effect on consumers’ purchase intention. Additionally, perceived behavior control has a significant mediating effect between consumers’ subjective norm and purchase intention; the consumption environment has a significant positive moderating effect on individual consumption intention. The study suggests that sustainable development can be further propelled by means of coordinating the consumers’ awareness of ecological environment protection and their enhanced consumption intention.

## Introduction

The COVID-19 dramatically changed the regular norms of society, international commodity trade, and consumption behavior. The culinary sector is no exception, and the pandemic causes the culinary sector labor shortage as well as the shutdown of factories, which affected the whole supply chain (Galanakis et al., 2021). As a highly export-oriented economy, the marine economy is characterized by remarkable openness, internationality, and globalization, which stimulates individuals’ demand for marine natural products and services, as well as demand and utilization intensity of marine resources (Gao et al., 2017). Marine aquatic products, as the basic carrier and main form of marine economy, occupy an important proportion in international trade exchanges, listed as the key supportive project for international trade exchanges. Therefore, it is imperative for operators and policymakers to effectively manage the turbulent business environment and consumption decisions following COVID-19.

The Food and Agriculture Organization of the United Nations has predicted that in the next decade, global per capita aquatic product consumption will continue to increase, and the total amount of aquatic product trade may increase significantly. At the same time, with the enhancement of individuals’ awareness of environmental protection, consumers’ demand for marine aquatic products is no longer limited to the product itself; instead, the purchase preference begins to swing between environmental protection and consumption upgrading, and environmental concerns play a partial mediating role between green marketing strategy orientation and green consumption behaviors (Ren et al., 2018; Khan et al., 2021). The impacts of COVID-19 on the culinary industry tend to last longer than we initially anticipated. Therefore, studying the coordinated development of the marine economy and ecological environment is significant for marine aquaculture enterprises to explore the international marketing pathways following the COVID-19 pandemic.

The literature involves the main theory of green marketing, which originates from the related research of Henion (1972) which defines the activities that are attributive to environmental problems and all measures that contribute to addressing environmental problems as green marketing (Groening et al., 2018). It advocates green environmental protection and green consumption, emphasizes the core value of economic benefits and ecological protection, and provides a theoretical basis for exploring the international marketing paths of marine aquatic products. However, the rational cognition of green consumption has not yet been established, and most enterprises have not yet defined the concept of green marketing. With the deepening of people’s understanding of ecological and environmental protection, green marketing has become a crucial topic in the current academic research. The research on green marketing mainly focuses on three aspects. First, the research on the connotation of green marketing stresses that green marketing is a management model that is identifiable, meets the social needs of consumers, and brings profits and sustainable operation (Shil, 2012; Kumar et al., 2013; Dangelico and Vocalelli, 2017). Second, the analysis of the challenges faced by the enterprises in developing green marketing primarily focuses on the challenges of marketing concepts, production methods, and marketing strategies (Kalafatis et al., 1999; Groening et al., 2018; Li et al., 2018). Third, the research on the green marketing measures implemented by the enterprises argues that the enterprises can establish a green brand through environmental innovation, green labels, trademarks, and effective environmental management systems to reflect their ecological advantages, appeal to the consumers of green products and stimulate their consumption intention (Chen et al., 2020; Azadnia et al., 2021; Khan et al., 2021).

Existing research in shopping response during the COVID-19 has focused mainly on customers’ multichannel (such as online or offline) shopping decisions, including hedonic motivation, shopping durations, and cross-cultural situations (Wang J et al., 2020; Wang S. et al., 2020; Li et al., 2021a,b; Wang M. et al., 2021). With the spread of COVID-19, the development of green marketing urgently requires the joint efforts of the government, enterprises, and consumers. However, few studies have considered the coordinated development between ecological protection and consumption upgrading (Waheed et al., 2020). Therefore, identifying the strategic positioning and choosing an ecologically oriented marketing path or a consumption-oriented marketing path will become an urgent issue for transnational marine aquaculture enterprises to consider in the future. This study mainly discusses the international green marketing paths of marine aquatic products to clarify the affective and cognitive characteristics of consumers between environmental protection and consumption upgrading, as well as the formation mechanism of environmental protection behavior. Moreover, this study proposes the concept of empathy marketing to further explore the psychological laws of green marketing and systematically analyzes the customer characteristics that empathize with marine aquatic products.

## Literature Review and Research Hypotheses

### Concept of Empathy and Marketing Applications

Empathy has been a key concept in clinical psychology theory and practice since it was introduced into the field of psychotherapy, and Rogers (1958) systematically elaborated the psychological, affective, and behavioral characteristics of applying empathy to cognitive psychotherapy (Yi et al., 2021), but the definition of empathy has not been unified in the existing literature. Some researchers believe that as an unconscious and involuntary emotion that is compatible with the object’s emotion (Abramson et al., 2020), empathy is the main driving force for prosocial behavior, and it can build links between one’s own and others’ emotional experiences and well-being (Marshall et al., 2020), playing an essential role in interpersonal communication and experience. Other researchers believe that empathy relies on the interplay between trait abilities and state influences (Brett and Maybery, 2021), describing it as the ability to understand and empathize with the experiences of others, and endowing it with “primary cognitive attributes (rather than affective attributes), including the ability to understand (rather than feel) the experiences of others and to convey such understanding and the intention to assist” (Schurz et al., 2021). The above viewpoints suggest that empathy is not only a simple cognitive structure or emotional phenomenon but a combination of cognitive empathy and affective empathy. It refers to empathizing with others and taking further concrete actions based on perceiving, recognizing, and understanding others’ thoughts and actions (Yoo and Whang, 2020; Jiang et al., 2021).

With the advent of the consumption era, the importance of empathy in the commercial field has become increasingly prominent, and it has gradually been integrated into the commercial market system. Zerbini et al. (2019) report that enterprises should take consumers’ emotions and needs as the core of their marketing strategies and achieve their strategic goals through cognitive and affective marketing activities. Its core idea is to establish empathy with consumers and clients to make the formulation and application of marketing strategies more flexible. Therefore, the marketing positioning of the enterprises needs to clarify the emotional and cultural connotation between the enterprises and their target customers, which is the basis of empathy marketing of the enterprises (Wang et al., 2019).

### Green Marketing

The rise of green marketing stems from the deterioration of the ecological environment and the enhancement of consumers’ awareness of environmental protection. It is a kind of business model based on green technology, green market, and green economy, responding to the ecological concerns of human beings (Dangelico and Vocalelli, 2017). The core of its strategy is to select and determine the marketing mix according to the principles of environmental protection and ecology. Green marketing is an extension and expansion of traditional marketing. It has both the commonality of general marketing and its own particularity. Compared with traditional marketing, green marketing is advanced, reasonable, and highly adaptable. It is a positive business management model, permeated with the concept of moral management, presenting new content different from the past. As a new trend in marketing, enterprises should highlight the ecological environment protection of the earth, promote the coordinated development of economy and ecology, make and implement plans related to products, prices, sales, and promotion to realize their own interests, consumer interests, social benefits, and ecological and environmental benefits (Chen and Yang, 2019; Li et al., 2022). It is a development process that an organization strives to incorporate environmental considerations into all its products and services and implement them in its marketing strategy through price promotion and distribution (Hong et al., 2020). The contents of green marketing include establishing green marketing concepts, collecting green information, formulating green strategies, developing green products, expanding green production, promoting green packaging, determining green prices, selecting green channels, conducting green promotions, strengthening green marketing services, guiding green consumption, implementing green management, etc. (Liu et al., 2021), and its core purpose is to reduce damage to the environment by adjusting measures such as product development and production processes, and gain the consumers’ acknowledgment (Gkargkavouzi et al., 2019) while allowing the consumers to choose their products and services based on certain environmental attributes of their products (Yenipazarli and Vakharia, 2017). The academic community believes that the government, enterprises, and the public are all contributors to green marketing and sustainable development, and it is indispensable to establish a mechanism for the government, enterprises, and the public to jointly manage and perform their own duties to develop green marketing (Huang et al., 2020).

### The Effect of Empathy Marketing on Purchase Intention

Empathy, the ability to understand others’ views and feelings, is an individual’s emotional response based on understanding others’ emotional states or circumstances. Pedersen (2021) claimed that as the pillar of marketing, empathy emphasizes and understands the importance of empathizing with consumers and customers to better implement marketing strategies. However, current marketing is increasingly pursuing objectification and specialization (Zerbini et al., 2019), resulting in a widening of the distance and fragmentation of marketing relations, and the empathy marketing method is indispensable to adjust. Empathy marketing originally implied that the enterprises inject brand concepts into the cognition and consumption behavior of target customers or consumers through empathy. In the face of differentiated interests, it is necessary to implement effective and personalized interaction strategies according to the characteristics of the customers and narrow the psychological distance between different interest groups (Brooks et al., 2011).

Purchase intention is the psychological performance of the consumers when they find a commodity that meets their own needs, which is the prelude to the purchase behavior and plays a decisive role in consumers’ purchase decisions (Kytö et al., 2019). In traditional shopping scenarios, the research on consumers’ purchase intention mainly concentrates on the consumers’ attitudes, maximization of consumers’ perceived value, and minimization of consumers’ perceived risk (Chen et al., 2019). In the research on consumers’ purchase intention, scholars have verified that in addition to the influence of consumer characteristics (such as age, income, occupation, etc.), product characteristics, and service quality, empathy in marketing is also a vital factor affecting consumers’ purchase intention. Empathy marketing triggers the consumers’ affective response by extending the brand concept of the enterprise into the cognition of the target customers or consumers, and the affective and cognitive responses constitute the motivation of consumers’ purchase intention (Wang W. et al., 2020, 2021). To sum up, the enterprises may empathize with the consumers through empathy marketing to trigger their purchase intention. In view of this, this study proposes the following hypothesis:

*H1: Empathy marketing has a significant positive effect on consumers’ purchase intention.*

### The Effect of Subjective Norm on Purchase Intention

The Theory of Planned Behavior (TPB) believes that the individual’s subjective norm, perceived behavior control, and behavioral attitude affect behavioral intentions and further affect the individual’s actions (Ajzen, 1991). Modeling and measuring the non-linear effects of underlying constructs, as well as explaining interactions and quadratic terms, scholars have verified the hypothesis that attitudes and subjective norms can predict application intentions (Titah and Barki, 2009). Wang S. et al. (2020) adopted the TPB model to divide attitudes into empirical attitude and instrumental attitude, subjective norms into normative social influence and informational social influence, and the perceived behavior control into product knowledge and perceived risk while considering the past experience and the interaction between the attitudes and subjective norms. Much research shows that the interaction effect of experiential attitude and subjective norm may positively affect the purchase intention. Therefore, this paper proposes the following hypothesis:

*H2: Subjective norm has a significant positive effect on the consumers’ purchase intention.*

### The Mediating Role of Perceived Behavior Control

Human behavior is not entirely voluntary but under control. Perceived behavior control refers to the individual’s perception of the difficulty of participating in a certain behavior, and it is an individual factor that affects behavioral intention. According to the research on the influence of perceived behavior control on behavioral intention, scholars have adopted the extensible theoretical model of planned behavior to explore the consumers’ purchase intention of green furniture and found that perceived behavior control has the greatest effect on consumers’ purchase intention, followed by health awareness, payment intention and subjective norms (Xu et al., 2020). Meanwhile, perceived behavior control is also a prerequisite for behavioral attitudes and subjective norms to influence individual behavioral intentions. If perceived behavior control is low, subjective norms and behavioral attitudes have relatively little effect on intention (Umeh and Patel, 2004). It is indicative that perceived behavior control plays an important role in predicting behavioral intentions, and it can also change the motivation of individuals’ behavioral intentions (Girelli et al., 2016; Ru et al., 2018). When consumers believe that they can easily participate in green consumption, they will increase their purchase intention of related products, and then perform the purchase behavior. Thus, this study proposes the following hypotheses:

*H3: Perceived behavior control has a significant positive effect on consumers’ purchase intention.*

*H4: Consumers’ subjective norm has a significant positive effect on perceived behavior control.*

### The Moderating Effect of Consumption Environment

With the continuous transformation and upgrading of people’s consumption needs and consumption structure, it is of great practical significance to find out the problems and weaknesses in consumption and create a harmonious and favorable consumption environment. The success of environmental innovation and sustainable transformation depends largely on the formation and diffusion processes of the market (Losacker and Liefner, 2020). Due to COVID-19, health awareness, environmental awareness, and social influence were identified as the main drivers of consumers’ purchase intention response (Qi et al., 2020; Zhang et al., 2020; Truong and Truong, 2022). Therefore, scholars have adopted the structural equation modeling method to explore how consumers’ previous purchase experience affects their environmental awareness and purchase intention of green products, highlight the perspective of consumers’ previous purchase experience of green products, expand consumer behavior research, and analyze the impacts of environmental awareness on purchase intention, and it is found that the influence of previous purchase experience and environmental awareness on individual purchase intention of green products depends on the purchase situation and product characteristics (Costa et al., 2021).

In short, the consumption environment affects the efficacy and effect of the combination of consumers and consumer goods. The consumers’ purchase intention is largely affected by product attributes and the consumption environment. With the enhancement of consumers’ awareness of environmental protection and the popularization of green marketing concepts, the green consumption environment created by society and the green attributes of the products play an increasingly important role in consumers’ purchase decisions (Wang S. et al., 2020). Based on this, this study proposes the following hypothesis:

*H5: The consumption environment has a significant positive moderating effect between the green marketing of the enterprises and the consumers’ purchase intention.*

**FIGURE 1:**
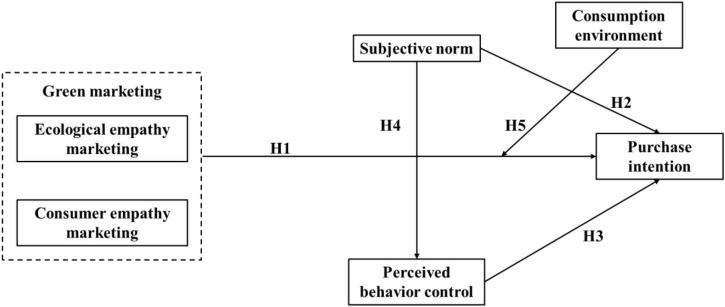
Theoretical model diagram.

According to the above research hypotheses, the theoretical model diagram of the green marketing path of marine aquatic products is constructed. Figure 1 shows the empirical quantitative research framework.

## Research Design

### Variables and Data Source

To comprehensively understand the international green marketing paths of marine aquatic products to clarify the affective and cognitive characteristics of consumers between environmental protection and consumption upgrading following COVID-19. This study adapted the Dispositional Empathy with Nature Scale formulated by Tam (2013) which has only one dimension and a total of 10 items. Ghasemi and Kyle (2021) also adopted the scale for the empathy for wild animals, but modified the wording of the original items and replaced the original items’ theme, animals and plants, with wildlife, and the reliability of each item was basically around 0.8. In this study, the context of “consumption of aquatic products” was added to the scale, and a 5-point scoring method was adopted.

This study also adapted the Customer Characteristics Scale developed by Homburg and Stock (2005) which consists of three dimensions, e.g., customer trust, price awareness, and the importance of products or services, with a total of 15 items. Abney et al. (2017) applied this scale to explore the effect of adaptive service restoration strategies through social media, the internal consistency coefficient was greater than 0.7, and each item showed an acceptable level of reliability. This study chose the customer trust dimension to measure empathy marketing.

The attitude variable of Ajzen’s TPB examined an individual’s attitude toward a behavior; the subjective norm variable includes characteristic attributes of an individual’s social environment; the perceived behavior control variable addresses changes in an individual’s ability to control behavioral performance. Cordano and Frieze (2000), based on Ajzen’s TPB, adopted two pollution prevention analyses to support and develop relevant research models and formulated measurement methods for relevant variables, in which the reliability of the subjective norm and perceived behavior control items were 0.74 and 0.79, respectively, with strong reliability and validity, and identified strong links between attitudes and behaviors related to sustainable development practices (Garay et al., 2019). It has been revised according to the topic of this study.

Additionally, this study adapted the Purchase Intention Scale proposed by Dodds et al. (1991) which has 6 items in total and adopts the 5-point Likert scale, with a reliability above 0.9. The perceived value plays an important role in consumers’ purchase decision making, and consumers choose specific products with higher perceived value (Yadav and Pathak, 2017). In line with the topic of this study, it was applied to measure the consumers’ purchase intention of green aquatic products.

The environmental consumer research explored an individual’s belief or feeling about a decision to purchase environmentally friendly products and the effect of this specific behavior on ecological consequences. However, the attitudes toward green products are different from general environmental attitudes, and this study adopted the Environmental Concern Scale formulated by Bamberg (2003), with a reliability above 0.8 (Jaiswal and Kant, 2018) and the 5-point Likert scale.

### Sample Selection and Data Collection

Since the outbreak of COVID-19 in December 2019, a number of pneumonia cases of unknown cause with a history of exposure to seafood markets in South China have been detected in some hospitals in Wuhan, Hubei Province, which have been confirmed as acute respiratory infections caused by Novel Coronavirus 2019 infection. The epidemic crisis implies opportunities for development (Yi et al., 2022). In the post COVID-19 era, the green and efficient aquaculture mode has gained more popularity, the modernization of fishery has been accelerated, and the high-quality development of aquaculture has ushered in a window period, pointing out the direction for ensuring the stable development of aquatic products, promoting green aquaculture technology, and innovating business forms and modes. Therefore, this study selected December 1, 2021 to December 17, 2021 as the period of time to explore the construction of green marketing path for aquatic products in the post COVID-19 era.

Before the formal survey, this study conducted a pre-survey to randomly selected consumers of different ages, which collected 42 questionnaires and analyzed the questions and suggestions of the respondents and the correlation and completeness of the data. In line with the principles of purpose, brevity, clarity, and rationality of the questionnaire, the content and form of the questionnaire were finally determined after some deletions were made to the questions.

The formal questionnaire survey consisted of two parts, one is the basic demographic information as the control variable, and the other is the scales of the five variables. The survey was primarily conducted online. To ensure the filling quality of the questionnaire, the respondents were informed before the survey that the questionnaire was only used for academic research and it would be kept confidential to eliminate their concerns. A total of 463 questionnaires were acquired. After eliminating random answers, missing questions, and inconsistent invalid questionnaires, 407 valid questionnaires were finally retained, with an effective recovery rate of 87.90%, which met the research requirement. The basic information of the samples is shown in Table 1. The basic attributes include: The numbers of male and female respondents are basically the same; Respondents aged 19–25 is the largest (28.7%); The number of the respondents with a junior college education or a bachelor’s degree is the largest (59.0%). Since the majority of the respondents of the survey were students, and result in the largest of the respondents with monthly disposable income below 2,000.

**TABLE 1:** Sample data and distribution.

Demographic variables		Count	Percentage%
Gender	Male	204	50.1
	Female	203	49.9
Age	Aged 18 and below	48	11.8
	Aged 19–25 (including 25)	117	28.7
	Aged 26–30 (including 30)	67	16.5
	Aged 31–40 (including 40)	69	17.0
	Aged 41–50 (including 50)	43	10.6
	Aged 51 and above	63	15.5
Educational background	Junior high school and below	76	18.7
	Senior high school, secondary school or vocational school	91	22.4
	University or Junior college	126	31.0
	Postgraduate or above	114	28.0
Occupation	Governmental department staff	58	14.3
	General worker or service personnel	63	15.5
	Business management personnel	43	10.6
	Engineer and technician	55	13.5
	Personnel in scientific research, education, and environmental hygiene	41	10.1
	Self-employed	43	10.6
	Other	104	25.6
Monthly disposable income	RMB 2,000 or less	106	26.0
	RMB 2,001–4,000 (including 4,000)	70	17.2
	RMB 4,001–6,000 (including 6,000)	66	16.2
	RMB 6,001–8,000 (including 8,000)	52	12.8
	RMB 8,001–10,000 (including 10,000)	60	14.7
	RMB 10,001 or above	53	13.0

## Research Findings

**FIGURE 2:**
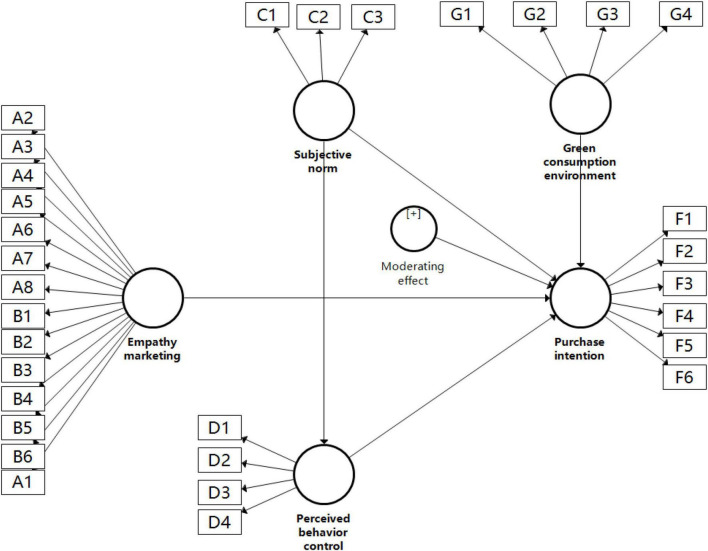
Preliminary model diagram.

The advanced data analysis techniques provide accurate results with the lowest error rate. Researchers are working on advanced research tools to improve the accuracy of data analysis. Based on structural equation modeling, PLS is also one of the advanced research techniques used in the analysis. Most researchers recommend the PLS-SEM technique for data analysis (Jermsittiparsert et al., 2019). Therefore, this study adopted PLS-SEM for data analysis, and the preliminary model diagram is shown in Figure 2.

### Measurement Model Analysis

**FIGURE 3:**
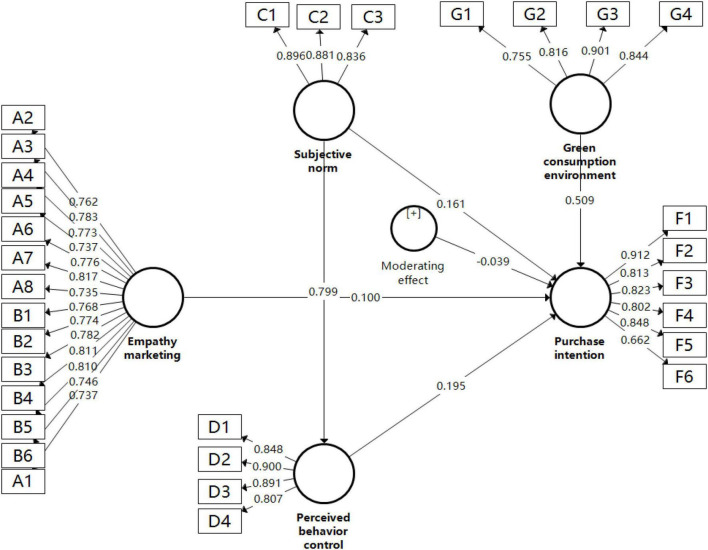
Measurement model analysis.

In terms of external model load analysis, as shown in Table 2 and Figure 3, the distribution of factors in each dimension conforms to the expected settings of the scale. To ensure the reliability of the data, the required indexes for the components are all above 0.65, the output results show that the data structure is reasonable, and the five required indexes have all received favorable feedback.

**TABLE 2:** External model load.

		Item	Score
Ecological empathy marketing	A1	When I consume aquatic products, I can imagine how I would feel if I were these plants and animals	0.737
	A2	When I consume aquatic products, I can immerse myself in the feelings of these plants and animals	0.762
	A3	When I consume aquatic products, I feel like I were just these plants and animals	0.783
	A4	When I consume aquatic products, I easily put myself in the situation of these plants and animals	0.773
	A5	When I consume aquatic products, the tough situation of these plants and animals will come clear to my mind	0.737
	A6	When I consume aquatic products, I will worry about these plants and animals	0.776
	A7	When I consume aquatic products, I can empathize how these plants and animals feel	0.817
	A8	When I consume aquatic products, I can empathize the pain these animals and plants suffer	0.735
Consumer empathy marketing	B1	We largely trust in marine aquaculture enterprises	0.768
	B2	We are sure that marine aquaculture enterprises keep their commitment to us	0.774
	B3	We believe that marine aquaculture enterprises are fair and honest to us	0.782
	B4	We believe that the information provided by the employees of marine aquaculture enterprises is correct	0.811
	B5	We are convinced that marine aquaculture enterprises accurately provide the green products/services	0.81
	B6	We believe that marine aquaculture enterprises will pay attention to our maximal interests	0.746
Subjective norm	C1	Those who are important to me in my life think that marine aquaculture enterprises should conduct green brand marketing	0.896
	C2	I think the government should take stronger action to protect our country’s resources	0.881
	C3	I think that the anti-pollution laws should be further enforced	0.836
Perceived behavior control	D1	Whether I buy more aquatic products is within my control	0.848
	D2	I have the right to change my consumption patterns when necessary to prevent pollution	0.900
	D3	I can access the resources needed to increase the number of green consumptions	0.891
	D4	Others support me to have green consumption	0.807
Purchase intention	F1	It is likely for me to buy green products and services	0.912
	F2	If I want to buy aquatic products, I will consider whether they are green products	0.813
	F3	I will consider whether to buy it at the price of the green product	0.823
	F4	It is of high possibility that I would consider buying green aquatic products	0.802
	F5	I’d like to buy green products and services provided by marine aquaculture enterprises	0.848
	F6	I think that green consumption is worthy of affirmation	0.662
Green consumption environment	G1	The newspaper or television reports about environmental issues make me angry	0.755
	G2	If we buy green products, we can further protect the environment	0.816
	G3	It is true that environmental protection still needs to be strengthened	0.901
	G4	We should buy green products for the sake of environmental protection	0.844

In the model testing process, the *R*^2^ value of perceived behavior control is 0.638, and the corrected *R*^2^ value is 0.637; the *R*^2^ value of purchase intention is 0.772, and the corrected *R*^2^ value is 0.769, indicating that each latent variable has a favorable explanatory effect on perceived behavior control and purchase intention.

In terms of construct reliability and validity analysis, standardized results were applied for analysis, as shown in Table 3. As for subjective norm, its Cronbach’s Alpha is 0.844, rho_A is 0.869, composite reliability (CR) is 0.904, and average extracted variance (AVE) is 0.759; as for empathy marketing, its Cronbach’s Alpha is 0.950, rho_A is 0.963, CR is 0.954, and AVE is 0.597; as for perceived behavior control, its Cronbach’s Alpha is 0.884, rho_A is 0.884, CR is 0.920, and AVE is 0.743; as for green consumption environment, its Cronbach’s Alpha is 0.849, rho_A is 0.851, CR is 0.899, and AVE is 0.690; as for purchase intention, its Cronbach’s Alpha is 0.895, rho_A is 0.899, CR is 0.921, and AVE is 0.662. The Cronbach’s Alpha coefficient of each latent variable is greater than 0.8, indicating that each latent variable has strong reliability; the CR is greater than 0.7, which further proves that the reliability of the model is high; and the AVE of each latent variable is greater than 0.5. From the above analysis, the overall fitting effect of the model is excellent, the potential internal relationship has a significant explanatory effect, the estimated results are acceptable, and the reliability indicators are consistent with the construct validity.

**TABLE 3:** Construct reliability and validity of the model.

	Cronbach’s alpha	rho_A	CR	AVE
Subjective norm	0.844	0.869	0.904	0.759
Empathy marketing	0.950	0.963	0.954	0.597
Perceived behavior control	0.884	0.884	0.920	0.743
Green consumption environment	0.849	0.851	0.899	0.690
Purchase intention	0.895	0.899	0.921	0.662
Moderating effect	1.000	1.000	1.000	1.000

### Structural Model Analysis

The Bootstrapping method is used to calculate the T statistic of each path coefficient, and the specific parameters are shown in Table 4 to test the significance level of the path coefficient estimate (two-tailed test). Among them, if 1.96 < T < 2.58, the path coefficient is significant at the 0.05 level; if 2.58 < T < 3.29, it is estimated to be significant at the 0.01 level; if T > 3.29, it is significant at the 0.001 level. The T statistic of the structural equation model in the Bootstrapping test shows that all path coefficients have a high T statistic, and the *P*-value of each path is less than 0.05, indicating that each path coefficient has passed the test of the corresponding significance level and the model has strong structural stability (Streukens and Leroi-Werelds, 2016).

**TABLE 4:** Path coefficients of the model.

	Original sample (O)	Sample mean (M)	Standard deviation (STDEV)	T statistic (| O/STDEV|)	*P*-value
Subjective norm - > Perceived behavior control	0.799	0.800	0.020	39.666	0.000
Subjective norm - > Purchase intention	0.161	0.159	0.046	3.516	0.000
Empathy marketing - > Purchase intention	0.100	0.101	0.035	2.830	0.005
Perceived behavior control - > Purchase intention	0.195	0.195	0.070	2.784	0.005
Green consumption environment - > Purchase intention	0.509	0.510	0.043	11.808	0.000
Moderating effect - > Purchase intention	–0.039	–0.038	0.017	2.318	0.020

In the meantime, to further verify the international green marketing paths of marine aquatic products, this study used the Bootstrapping method to verify the path coefficient of specific indirect effects. The coefficient of the original sample (O) is 0.156, the coefficient of the sample mean (M) is 0.156, the standard deviation (STDEV) coefficient is 0.056, the T statistic is 2.768, above 2.58, and the *P*-value of each indirect path is less than 0.01. The results prove that the specific indirect effect in the model has passed the test of the corresponding significance level and has a favorable specific indirect effect performance.

### Hypotheses Test

The H1, H2, H3, and H4 hypotheses are validated in the tests above. First, Table 4 suggests that empathy marketing has a significant positive effect on consumers’ purchase intention, and the path coefficient is 0.100, indicating that empathy marketing can inject the brand proposition of the enterprise into the cognition of target customers or consumers, and adopt emotional and personalized interaction strategies to shorten the psychological distance with different interest groups so that consumers are encouraged and stimulated to purchase, therefore H1 is verified. Second, Table 4 suggests that subjective norm has a significant positive effect on consumers’ purchase intention, and the path coefficient is 0.161, indicating that consumers tend to be influenced by important people around them. They are more willing to purchase green products when important people around them think it is better to purchase green products, thus H2 is verified. Third, Table 4 suggests that perceived behavior control has a significant mediating effect between consumers’ subjective norm and purchase intention. Particularly, perceived behavior control has a significant positive effect on consumers’ purchase intention, and the path coefficient is 0.195, hence H3 is verified, and the subjective norm has a significant positive effect on perceived behavior control, and the path coefficient is 0.799, thus H4 is verified. Combined with the significant specific indirect effects, perceived behavior control acts as a mediating factor between subjective norm and purchase intention, which proves that given that consumers can get corresponding social support and the purchase of green products is within their control, they have greater intention to purchase green aquatic products.

### Moderating Analysis

**FIGURE 4:**
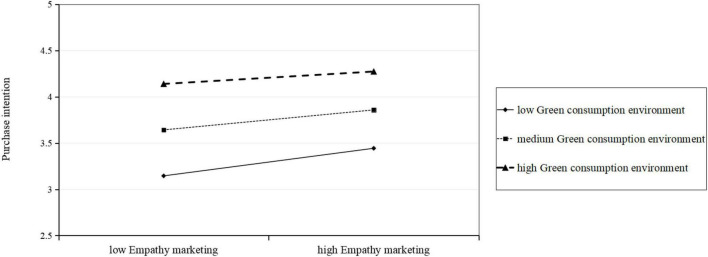
Moderating effect analysis of the model.

First, the variables related to the moderating effect are set up, respectively, the dependent variable is purchase intention, the moderating variable is green consumption environment, and the independent variable is empathy marketing. The Bootstrapping method is adopted to calculate the T statistic of each path coefficient. As seen in Table 4, the *T* value of the interaction item between empathy marketing and green consumption environment is 2.318, and the *P*-value is less than 0.05, indicating that the interaction effect is significant, and the green consumption environment plays a moderating role in empathy marketing and purchase intention. To more intuitively reflect the moderating effect of green consumption environment in empathy marketing and purchase intention, the green consumption environment plus or minus one standard deviation is used as the benchmark, and the effects of empathy marketing on purchase intention under different green consumption environments are made clear. The effect patterns of this interaction are illustrated in Figure 4.

As shown in Figure 4, whether in a low green consumption environment or a high green consumption environment, the regression slope of empathy marketing to consumers’ purchase intention is positive. Therefore, the green consumption environment plays a positive moderating role in the effect of empathy marketing on purchase intention.

The regression slope in the low green consumption environment is slightly higher than that in the high green consumption environment, indicating that compared with the high green consumption environment, high-level empathy marketing will have more positive effects on consumers’ purchase intention in the low green consumption environment. Based on the above analysis, the green consumption environment has a significant positive moderating effect between empathy marketing and purchase intention, which verifies H5.

## Research Conclusion

### Conclusion

With the increasingly frequent economic and trade exchanges between countries in the world and the continuous expansion of international commodity trade (Duan et al., 2022), marine aquatic products are the basic carrier and main manifestation of the marine economy. Meanwhile, the prolonged COVID-19 pandemic constantly improves consumers’ environmental protection awareness. The consumers’ demand for marine aquatic products is not only limited to the products *per se*, but also swings between environmental protection and consumption upgrading. Therefore, it is urgent for the marine aquaculture enterprises to explore appropriate international marketing paths and psychological laws of green marketing from the pro-environment perspective.

This study attempted to construct green marketing paths for the marine aquatic products following the COVID-19 pandemic. In previous research on green marketing, scholars have mainly focused on the research on the connotation of green marketing, the challenges faced by the enterprises in carrying out green marketing, and the measures for the enterprises to implement green marketing (Chen et al., 2020), but rarely considered the coordination between ecological protection and consumption upgrading, in other words, the effects of ecological empathy marketing and consumer empathy marketing on purchase intention are rarely addressed. It is found in this study that empathy marketing (including ecological empathy marketing and consumer empathy marketing) has a significant positive effect on consumers’ purchase intention, and the green consumption environment plays a positive role between empathy marketing and purchase intention. A favorable green consumption environment contributes to evoke consumers’ experience of the natural environment and trigger a higher degree of empathy between consumers and nature, which positively affects the consumers’ emotional brand attitude toward green aquatic products and ultimately makes them have more positive purchase intention. Moreover, the consumers’ subjective norm can positively influence consumers’ purchase intention through their subjective behavior control. Therefore, countries and enterprises should give priority to environmental protection when conducting international marketing of marine aquatic products, enhance the whole society’s awareness of environmental protection, enable the consumers to get the support of others when performing green consumption, and improve the green consumption quality in a bid to promote the consumers’ purchase intention. The extent to which green marketing can arouse empathy between consumers and nature is the key to the international trade of marine aquatic products. The stronger the empathy between consumers and nature, the more intense their emotional brand attitude and purchase intention.

### Theoretical Implications

In the post COVID-19 era, in the international marketing of marine aquatic products, creating a green brand is crucial for sustainable consumption. From the theoretical point of view, this study is of great significance as follows: on the one hand, it has extended the understanding of empathy. In previous studies, empathy is divided into cognitive empathy and affective empathy, which is mostly applied in the research of prosocial behavior, emotion recognition, nursing, etc. (Juhl et al., 2020; Levett-Jones and Cant, 2020; Brett and Maybery, 2021). This study has combined empathy and ecology to broaden the concepts of empathy marketing and green marketing and divide empathy marketing into ecological empathy marketing and consumer empathy marketing to enable the consumers to empathize with the environment and the enterprises with an aim to strengthen the coordination between ecological protection and consumption upgrading and expand the international marketing of marine aquatic products. On the other hand, this study revealed that improving the consumers’ subjective norm and perceived behavior control in green consumption can stimulate their purchase intention of green products and increase their pro-environmental behavior in that the public will not only adjust their environmental protection behaviors by directly observing other individuals’ behaviors but also gain environmental protection knowledge through social interaction, thereby exerting a positive effect on their behaviors. This study has once again verified the points of previous studies (Zheng et al., 2019), and contributed to the research in the field of sustainable development in the post-COVID-19 pandemic era.

### Practical Implications

This study also has the following practical implications. First, the findings show that empathy marketing (including ecological empathy marketing and consumer empathy marketing) and subjective norm can positively affect consumers’ purchase intention of green aquatic products. Therefore, while enhancing the green brand effect, marine aquaculture enterprises must also create a favorable green consumption environment, form strong awareness of environmental responsibility, apply social norms to trigger consumers’ awareness of norms, and improve the possibility of consumers choosing a green aquatic product to further increase consumers’ purchase intention of green products. Second, in the post COVID-19 pandemic era, competitive marine aquaculture enterprises serve as an effective information channel between consumers and original brand manufacturers to enable the consumers to better understand the relevant information and functions of green products and enhance the influence of green brands and positive attitudes of consumers toward green aquatic products, as well as consumers’ purchase intention of green brand products. Third, since consumers’ perceived behavior control of green aquatic products in this study has a significant mediating effect between their subjective norm and purchase intention, the enterprises may increase the consumers’ purchase intention of green products by improving their perceived behavior control.

### Research Limitations and Future Direction

This study contributes to facilitating managers to develop more effective green marketing strategies to drive sustainable consumption and urge the marine aquaculture enterprises to thoroughly understand the sources of the consumers’ purchase intention of green products of the post COVID-19 era. However, this study also has its weakness which needs further improvement in future research. First, since the respondents in the current study were selected from various industrial sectors in China who consume green aquatic products, their epidemic situation and cultural differences in various regions may also lead to different green consumption behaviors of consumers, thus affecting the relationship between variables in this study. Therefore, based on the theoretical model of this study, future studies may focus on comparing the path differences between empathy marketing of green aquatic products and consumers’ purchase intention in different countries or regions. Second, this study adopted the method of a questionnaire survey to test the hypothesis. Although we selected a period in the post COVID-19 era, only horizontal data was provided, making it difficult to observe the dynamic changes of empathy marketing, subjective norms, perceived behavior control, and purchase intention of green products in the COVID timeline. Future research may conduct longitudinal studies to understand the differences in empathy marketing, subjective norm, perceived behavior control, and purchase intention of green products in different periods. The findings of the current study are beneficial to stimulate more future research, provide feasible suggestions for the government, enterprises, and consumers to expand the international green marketing paths, coordinate the consumers’ awareness of ecological environment protection with enhanced consumption, and further improve the psychological laws of green marketing to make a comprehensive and systematic analysis of consumer characteristics that empathize with marine aquatic products of the post COVID-19 era.

## Data Availability Statement

The raw data supporting the conclusions of this article will be made available by the authors, without undue reservation.

## Ethics Statement

The studies involving human participants were reviewed and approved by Nanchang Institute of Science and Technology, China. The patients/participants provided their written informed consent to participate in this study. Written informed consent was obtained from the individual(s) for the publication of any potentially identifiable images or data included in this article.

## Author Contributions

LX contributed to the empirical work, the analysis of the results, and the writing of the first draft. YQW, YTW, and YZ supported the total work of LX. TM and QZ revised and supervised this manuscript. All authors contributed to the article and approved the submitted version.

## Conflict of Interest

The authors declare that the research was conducted in the absence of any commercial or financial relationships that could be construed as a potential conflict of interest.

## Publisher’s Note

All claims expressed in this article are solely those of the authors and do not necessarily represent those of their affiliated organizations, or those of the publisher, the editors and the reviewers. Any product that may be evaluated in this article, or claim that may be made by its manufacturer, is not guaranteed or endorsed by the publisher.
